# EP21 Tumour necrosis factor inhibitor for the treatment of refractory extra-pulmonary disease including sarcoid arthritis and myositis

**DOI:** 10.1093/rap/rkaa052.020

**Published:** 2020-11-03

**Authors:** Sanketh Rampes, Vishit Patel, Deepak Nagra, Jennifer Hannah, James Galloway

**Affiliations:** r1 Faculty of Life Sciences & Medicine, King’s College London, London, United Kingdom; r2 Academic Rheumatology Department, King’s College London, London, United Kingdom

## Abstract

**Case report - Introduction:**

Sarcoid myositis is a rare extrapulmonary manifestation of sarcoid, histologically characterised by non-caseating granuloma in the perimysial connective tissue. Muscle involvement is often asymptomatic but can cause weakness, myalgia, or muscle nodules. The first line treatment for sarcoid myositis is steroids. This case report details a case of sarcoid myositis and multi-system disease refractory to both steroids and mycophenolate mofetil. The use of infliximab, a Tumour Necrosis Factor α inhibitor (TNFi) resulted in drastic clinical and radiological resolution of sarcoid myositis. The patient is still on the TNFi and his sarcoid remains relatively well controlled.

**Case report - Case description:**

A 33-year old Afro-Caribbean gentleman with a 10-year history of sarcoid (biopsy confirmed, affecting lymph nodes, lung) presented in May 2017 with joint pain, weakness, difficulty walking and cognitive impairment. At the time of the original diagnosis of sarcoid, he had concurrently been diagnosed with schizophrenia and steroids had been avoided. His only medication was olanzapine 5mg.

Due to declining cognitive function, his psychiatrists arranged an MRI brain which showed extensive leptomeningeal disease. Lumbar puncture excluded infection and neurosarcoid was diagnosed. Prednisolone resulted in partial improvement in cognition, however joint symptoms and weakness persisted.

On rheumatologic assessment there was an asymmetric inflammatory arthropathy, with small joint synovitis as well as large volume joint effusions of elbows and knees. Radiologic investigations showed characteristic lattice bony destruction, consistent with osseous sarcoid. His gait was waddling, and muscle strength was reduced symmetrically in the proximal groups.

His creatinine kinase was 865, myositis specific antibody screen was negative although U1RNP was equivocal. Serum ACE was 102, CRP 17, ESR 25. CT-PET demonstrated FDG avidity in lymph nodes, lung parenchyma, bones, muscles, and testes. The muscle involvement was typical of nodular sarcoid and did not correlate with his weakness. The disease failed to respond adequately to either methotrexate or mycophenolate. He started Infliximab (5mg/kg). There was remarkable resolution of symptoms, including improvement in cognition. Follow up CT-PET confirmed response. Three years on he remains well, and no longer takes olanzapine.

**Case report - Discussion:**

Muscle involvement occurs in 50-80% of sarcoidosis patients. Symptomatic myositis is rare (0.5-2.5%). There are three types of sarcoid myositis: nodular form, chronic myopathy, and acute myositis. Nodular involvement, as seen in our patient, usually occurs in young adults who experience palpable, painless nodules which may occur in any muscle. Nodules are not usually associated with weakness or limitation of movement. EMG and CK are usually normal. Chronic myopathy is rarely observed. It is characterised by a slowly progressive, symmetrical proximal myopathy with myopathic EMG changes but normal muscle enzymes, usually in women aged 50-60. Acute myositis typically affects younger patients (<40 years old) with diffuse muscle swelling, pain and proximal weakness which may progress to hypertrophy and contractures. Fatigue, fever, joint symptoms, and erythema nodosum are frequently seen. Inclusion Body Myositis is another granulomatous myopathy and should be considered as a differential, particularly in cases of treatment failure.

The patient has had several episodes of psychosis and confusion which were previously diagnosed as schizophrenia and corticosteroid induced psychosis, meaning the team used steroids cautiously. MRI brain imaging revealed the presence of extensive neurosarcoidosis, and the neurocognitive improvement with treatment of a TNF inhibitor, suggested that the underlying pathology was sarcoidosis. Steroids could therefore be utilised appropriately for ongoing management. This case illustrates the difficulty of teasing out the underlying aetiology of neurocognitive dysfunction in patients with extensive sarcoidosis.

TNFα released by alveolar macrophages is implicated in the induction and maintenance of sarcoid granulomas. Limited data from small randomised controlled trials and increasing data from non-randomised studies have led to consensus-based recommendations for TNFi use in pulmonary, ocular, cutaneous, neurological, and multi-system sarcoidosis. To our knowledge, this case is the first documented example of rapid clinical and radiological resolution of sarcoid myositis with an anti-TNF agent.

**Case report - Key learning points:**

There are three important points to take away from this case.

First, this case highlights the importance of a PET scan in demonstrating multi-system involvement in sarcoidosis. The PET scan was key in highlighting the extent of disease including sarcoid myositis. MRI scans of the brain were also important in highlighting the extent of neurosarcoidosis. Second, the patient presented with psychosis in 2013. This was thought to be corticosteroid-induced at the time. However, since treatment with the TNF inhibitor the patient experienced a significant neurocognitive improvement. The drastic improvement undermines the diagnosis of primary psychosis and suggests that the psychosis may have been due to neurosarcoidosis. In the context of patients with multi-organ sarcoidosis, psychosis secondary to neurosarcoid should be considered as a differential, even in the context of an earlier diagnosis of schizophrenia pre-dating the diagnosis of sarcoid.

Third, the drastic resolution of sarcoid myositis and arthritis with a TNF inhibitor suggests that TNF inhibitors should be considered as treatment for cases of sarcoid myositis or sarcoid arthritis refractory to steroids. Whilst TNF inhibitors are currently unlicensed for this use in the UK, there is a growing body of evidence for their effectiveness in treating refractory extra-pulmonary sarcoidosis.

